# Bilateral Nephroblastoma in a Toddler: Hypertension as a Critical Diagnostic Clue

**DOI:** 10.7759/cureus.108536

**Published:** 2026-05-09

**Authors:** Nour El Houda Fakhri, Meriem Ajrhourh, Karima Ryouni, El Alaoui Mounia, Noufissa Benajiba

**Affiliations:** 1 Pediatrics, Abderrahim Harouchi Mother-Child Hospital, Casablanca, MAR; 2 Pediatric Hematology and Oncology, Abderrahim Harouchi Mother-Child Hospital, Casablanca, MAR; 3 Hematology and Oncology, Abderrahim Harouchi Mother-Child Hospital, Casablanca, MAR; 4 Pediatric Hematology, Centre Hospitalier Universitaire Mohammed VI Oujda, Oujda, MAR

**Keywords:** bilateral nephroblastoma, pediatric hypertension, renin-secreting tumor, status epilepticus, wilms tumor

## Abstract

Bilateral nephroblastoma, also known as Wilms tumor, represents a rare yet critical pediatric renal malignancy. Hypertensive emergencies with seizures are exceptional. We report the case of an 18-month-old girl who presented with status epilepticus secondary to malignant hypertension (180/120 mmHg) associated with a two-week history of abdominal distension. Clinical examination revealed bilateral lumbar masses, and computed tomography confirmed the presence of bilateral renal tumors, the largest measuring 102 × 80 × 100 mm. Neoadjuvant chemotherapy resulted in a partial response, with tumor size reduced to 69 × 60 × 63 mm, pending nephron-sparing surgery. Rapid diagnosis is crucial, as seizures presenting as the initial manifestation are rare and may delay recognition of the underlying abdominal pathology. Early imaging in cases of pediatric hypertension can prevent severe complications and improve outcomes, allowing renal preservation with survival rates exceeding 85%. Wilms tumor should be suspected in hypertensive toddlers presenting with abdominal signs, and prompt ultrasound evaluation is essential to avoid diagnostic delay and associated complications.

## Introduction

Bilateral nephroblastoma, also known as Wilms tumor, is a rare but important pediatric renal malignancy, accounting for approximately 5-10% of all Wilms tumor cases and typically manifesting in children under five years of age, as illustrated by the present case, with firm bilateral abdominal masses extending to the lumbar regions [1,2].

Hypertension is a pivotal diagnostic clue in up to 25-63% of Wilms tumor cases, primarily driven by tumor secretion of renin from intrarenal juxtaglomerular cells [3], leading to secondary hyperreninemic hyperaldosteronism. Clinicians must urgently consider nephroblastoma in any child presenting with new-onset hypertension accompanied by abdominal distension or a palpable mass, as diagnostic delays may precipitate life-threatening complications, such as encephalopathy, seizures, or cardiac strain [3,4,5]. Diagnostic confirmation relies on imaging, which further supports timely management and initiation of neoadjuvant chemotherapy [1,2,6].

## Case presentation

A one-year-and-six-month-old girl, with no significant past medical history, presented to the emergency room in February 2025 with an acute afebrile seizure. On history taking, it was revealed that the patient had abdominal distension evolving over the past two weeks. Examination revealed firm bilateral abdominal masses occupying the lumbar regions bilaterally, with malignant hypertension measuring 180/120, above the 95th percentile + 30 mmHg [7].

The patient was admitted to the pediatric intensive care unit and started on intravenous nicardipine (Loxen) continuous infusion for blood pressure stabilization (dose: 0.5-1 µg/kg/min), alongside oral antihypertensives after stabilization (amlodipine dose: 0.5 mg/kg/day, acebutolol dose: 5 mg/kg/day) and intravenous phenobarbital initially at a dose of 20 mg/kg IV slow infusion, followed by 5 mg/kg. 

Initial laboratory investigations showed microcytic anemia, normal white blood cell count with lymphopenia, and normal platelets, normal renal function, and elevated LDH (Table 1).

**Table 1:** Initial lab workup of the patient. Values in bold are the anomalies in the initial blood work of the patient. MCV: mean corpuscular volume, MCHC: mean corpuscular hemoglobin concentration, WBC: white blood cell, LDH: lactate dehydrogenase

Parameter	Value	Unit of measure	Reference range
Hemoglobin	9.1	g/dL	11.0-13.5 g/dL
MCV	68	f/L	70-86 f/L
MCHC	29	pg	31-36 pg
WBC	7630	/µL	6.0-17.5 x10^3^ /µL
Neutrophils	6300	/µL	1.5-8.5 x 10^3^/µL
Lymphocytes	700	/µL	2.0-9.0 x10^3^/µL
Platelets	391000	/L	150-450 x10^3^/L
Reticulocytes	2.2	%	0.5-2.5 %
Urea	0.15	g/L	0.09-0.38 g/L
Creatinine	2.4	mg/L	2-4 mg/L
LDH	650	U/L	160-450 U/L

**Figure 1:**
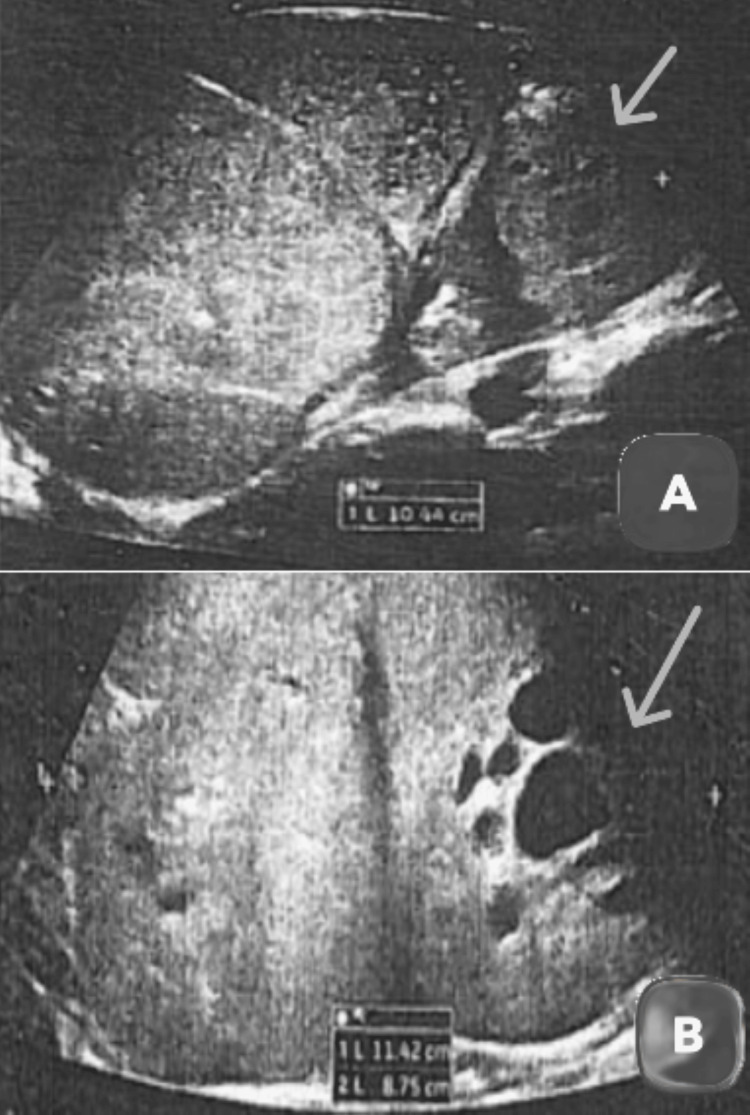
Initial abdominal ultrasound of the patient A: Enlarged left kidney with identification of multiple well-defined, rounded, heterogeneous isoechoic masses containing cystic areas, the largest measuring 104 × 78 mm in its greatest dimensions, as seen in the upper image. B: A large mass centered in the right hypochondrium and right flank, well-defined with lobulated contours, of heterogeneous echostructure, containing cystic areas, measuring 114 × 87.5 mm, as seen in the lower image.

Abdominal ultrasound demonstrated bilateral intra-abdominal masses arising from the kidneys with associated bilateral hydronephrosis (Figure 1).

**Figure 2:**
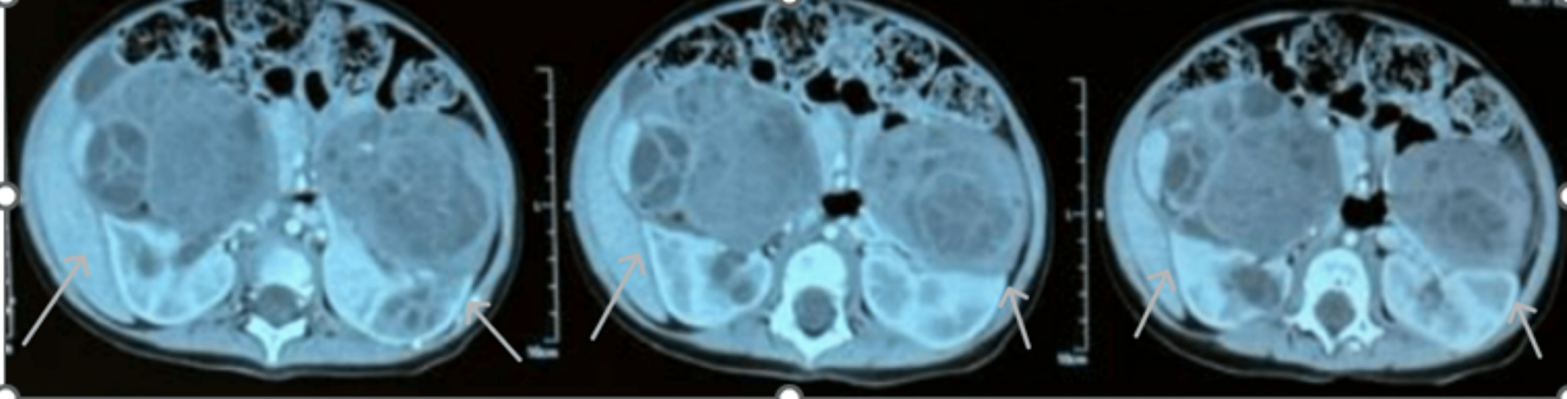
Initial abdominopelvic CT scan of the patient Bilateral renal masses, with three lesions on the right kidney and two on the left. The largest right upper- and mid-pole mass was well-circumscribed, lobulated, and hypodense, measuring 102 × 80 mm with a longitudinal extent of 100 mm. The left mid-renal mass had similar characteristics, measuring 91 × 64 mm with an extent of 83 mm. Additional smaller lesions were noted bilaterally: right upper pole 38 × 26 mm, right mid renal 17 × 12 mm, and left upper pole 43 × 42 mm.

Thoracoabdominopelvic CT scan on 24/02/2025 confirmed bilateral nephroblastoma comprising two left renal masses and three right renal masses (largest 102 × 80 × 100 mm), with incidental bilateral basal atelectasis and sequelae of lacunar lesions in the right lenticular nucleus and left internal capsule (Figure 2).

Diagnosis of bilateral nephroblastoma (stage V) was established, and neoadjuvant chemotherapy was initiated per the Groupe Franco-Africain d’Oncologie Pédiatrique (GFAOP) Nephro 2005 protocol. The patient was treated on a weekly alternating schedule: vincristine alone intravenously at a dose of 1.5 mg/m^2^ one week, followed by vincristine plus actinomycin D the following week; vincristine intravenously at a dose of 1.5 mg/m^2^ with actinomycin D intravenously at a dose of 45 µg/kg, achieving partial response on serial imaging (first evaluation 15/04/2025: 80 × 61 × 90 mm; second 29/05/2025: 69 × 60 × 63 mm) with ongoing preoperative therapy pending nephron-sparing surgery (with a minimum planned duration of 12 weeks, followed by radiological re-evaluation and therapeutic decision-making based on the treatment response).

## Discussion

Bilateral nephroblastoma remains an uncommon presentation, representing about 5-8% of cases. It typically occurs in very young children, with most series reporting a median age between 28 and 33 months. In that context, the diagnosis in our 18-month-old patient reflects an early but not unprecedented presentation [8,9]. In routine practice, an abdominal mass is the most frequent finding, reported in up to 90% of patients. However, hypertension is also well recognized, occurring in roughly 20-63% of cases, and is thought to result from increased renin secretion by tumor-associated juxtaglomerular activity. When present alongside abdominal distension, it should prompt immediate evaluation for a renal origin [10,11].

In our patient, the diagnostic process was complicated by the mode of presentation. Hypertensive emergencies revealing Wilms tumor are rare, and cases presenting with seizures remain exceptional, with only a limited number described in the literature. These neurologic manifestations are often attributed to posterior reversible encephalopathy syndrome in the setting of acute, severe hypertension [2,12]. Here, status epilepticus was the initial event leading to intensive care admission. This type of presentation can easily divert attention toward primary neurologic causes, delaying consideration of an underlying abdominal pathology. Such diagnostic distraction is well recognized, and autopsy data suggest that a small proportion of pediatric malignancies may go unrecognized during life when initial symptoms are misleading [2,12].

The clinical course in this case also illustrates the importance of early therapeutic strategy. Management followed the GFAOP Nephro 2005 protocol, derived from the International Society of Paediatric Oncology (SIOP) 2001 protocol approach, with preoperative chemotherapy aimed at tumor reduction and renal preservation. A clear decrease in tumor size was observed after treatment, which is consistent with previously reported responses to vincristine and actinomycin D. This reduction is critical in bilateral disease, where preserving functional renal tissue directly impacts long-term outcomes, and survival rates now exceed 85% in optimized settings [9,12].

From a management perspective, the combination of antihypertensive therapy targeting the renin-angiotensin system and chemotherapy allowed control of both blood pressure and tumor progression, avoiding immediate nephrectomy. Nevertheless, the risk of late complications remains significant. Children treated for bilateral disease are exposed to a substantial risk of chronic kidney disease and persistent or recurrent hypertension, which justifies prolonged follow-up [12].

Overall, this case highlights a practical point: severe hypertension in a young child, particularly when associated with abdominal findings, should not be attributed solely to primary cardiovascular or neurologic causes. Early abdominal imaging, especially ultrasonography, may allow timely identification of renin-secreting renal tumors and help prevent life-threatening complications such as those observed here.

## Conclusions

Bilateral nephroblastoma in young children is rare but should be considered in the presence of malignant hypertension with abdominal distension. Seizures may mask the underlying diagnosis and delay imaging. Early abdominal ultrasound is essential for prompt identification, allowing rapid initiation of chemotherapy and kidney-sparing management. Early recognition significantly improves outcomes and reduces life-threatening complications.
